# Metastatic bilateral adrenal sarcomatoid carcinoma: Evaluation by ^18^F-FDG PET/CT

**DOI:** 10.22038/AOJNMB.2021.57606.1400

**Published:** 2022

**Authors:** Swati Rachh, Patel Nilam

**Affiliations:** Department of Nuclear medicine, The Gujarat Cancer & Research Institute, New Civil Hospital Campus, Ahemdabad, India

**Keywords:** Adrenal sarcomatoid carcinoma, Spindle cell, ^18^F-FDG PET/CT

## Abstract

Sarcomatoid carcinoma of the adrenal gland is an uncommon presentation of malignant adrenal tumors and bilateral presentation is extremely rare. It is an extremely rare occurrence, unusual symptoms, and both epithelioid and sarcomatoid components in histology are a challenge to diagnose sarcomatoid carcinoma of adrenal origin. The majority of patients are diagnosed at a later stage while having metastatic disease and succumb due to disease within a few months of diagnosis due to the aggressive nature of the disease. Probably due to the advanced disease at the time of diagnosis; patients diagnosed having adrenal sarcomatoid tumor have a very poor prognosis. In nonmetastatic disease, adjuvant chemotherapy is suggested after the removal of the tumor. It is essential to diagnose these tumors earliest to treat with effective treatment modalities. The present study describes the rare case of sarcomatoid carcinoma involving the bilateral adrenal gland with metastasis to bones, lymph nodes, and pleura evaluated by ^18^F-FDG PET/CT.

## Introduction

 Adrenocortical carcinoma (ACC) is an uncommon, aggressive malignancy and sar- comatoid carcinoma- a rare morphological variant of ACC is a biphasic tumour with sarcoma like or sarcomatoid component and has a poor prognosis with rapid fatal behavior (1). Adrenal sarcomatoid carcinoma is a very rare malignant tumour and to date there are only 21 cases reported in the English literature (2) and to the best of our knowledge there has been only two cases with bilateral disease (3, 4). Here, we report a case presented with skeletal metastasis and was evaluated by Fluorine-18-Flurodeoxy-glucose positron emission tomography/ computed tomography (^18^F-FDG PET/CT) and IHC having sarcomatoid carcinoma involving the bilateral adrenal gland with metastasis to bones, lymph nodes, and pleura.

## Case Report

**Figure 1.1:**
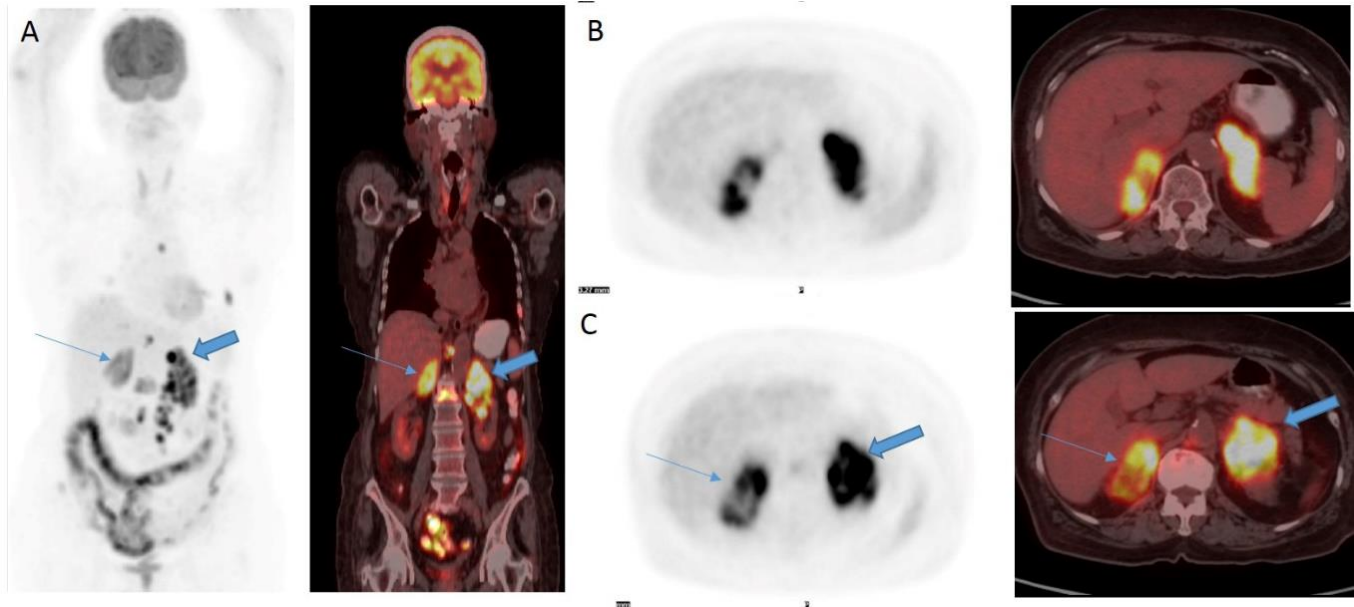
FDG PET (SUV range:0-5) whole body MIP image FDG PET/CT fused coronal (**A**) transaxial images (**B**), (**C**) revealed FDG avid soft tissue density lesion involving bilateral enlarged adrenal gland measuring 6.3×4.6 cm on the right side (**Thin arrow**), and 6.0×3.1 cm on the left side (**Thick arrow**)

 A 78 year old female patient without any significant comorbid conditions, presented to the research institute with severe back pain. MRI of lumbosacral spine revealed marrow edema and infiltration involving L1 vertebral body with no evidence of disc involvement or pre/ para-vertebral collection and bilateral bulky adrenal glands. Biopsy of L1 vertebra objectified atypical spindle cell lesion infiltrating marrow. The patient subjected to FDG PET/CT scan to look for primary lesion in view of the metastatic bony lesion. ^18^F-FDG PET whole body MIP image ^18^F-FDG PET/CT fused coronal (A) transaxial images (B) and (C) reveals FDG avid soft tissue density lesions involving bilateral enlarged adrenal gland measuring 6.3×4.6 cm on the right side (SUV_max_=25.3) [Thin arrow] and 6.0×3.1 cm on the left side (SUV_max_=10.5)[Thick arrow] Figure 1.1. 

 IHC of L1 vertebral lesion revealed SMA (smooth muscle) marker positive and CK (epithelial marker) positive in some cells with negative P-40, TTF-1, PAX-8, Desmin, CD31/CD34, CK7/CK20 markers which ruled out tumours of lung, thyroid, renal, ovary, rhabdosarcoma, vascular or epithelial origin.

**Figure 1.2:**
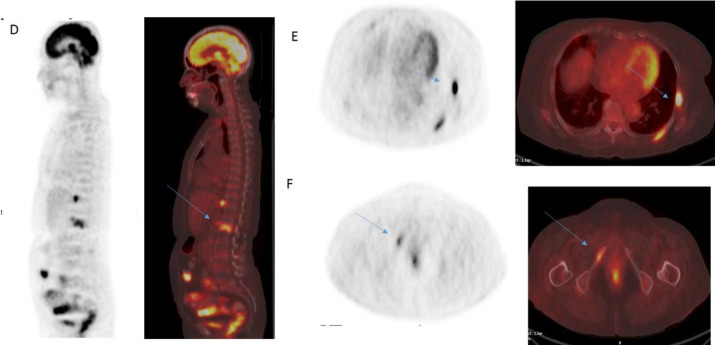
FDG PET (SUV range:0-4) and PET/CT fused sagittal image (**D**) revealed FDG avid lytic lesion involving L1 vertebral body [**Arrow**] and transaxial images revealed lytic lesions involving left sided ribs (**E**), and right inferior pubic ramus(**F**) [**Arrow**]

**Figure 1.3:**
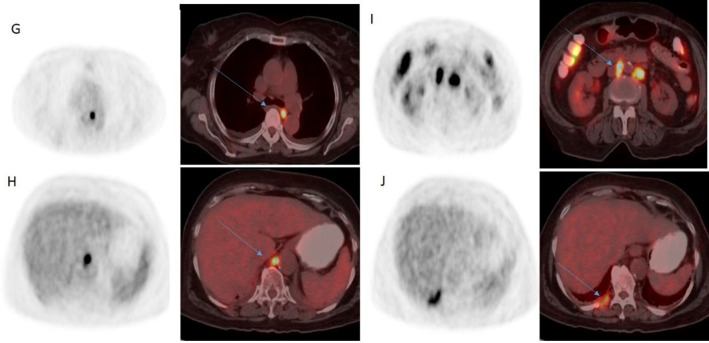
FDG PET (SUV range: 0-4) and PET/CT fused transaxial images revealed FDG avid mediastinal (**G**), retrocrural (**H**) and retroperitoneal (**I**) lymphnodes and pleural lesion. (**J**) [**Arrow**]

^18^F-FDG PET and PET/CT fused sagittal image (D) revealed FDG avid lytic lesion involving L1 vertebral body (SUV_max_=9.5), transaxial images revealed lytic lesions involving left sided ribs (SUV_max_=10.2) (E), and right inferior pubic ramus (SUV_max_=6.0) (F) Figure 1.2. ^18^F-FDG PET and PET/CT fused transaxial images revealed FDG avid mediastinal (SUV_max_=10.7) (G), retrocrural (SUV_max_=10.8) (H), retroperitoneal (SUV_max_= 14.5) (I) lymph-nodes and pleural lesion (SUV_max_=5.5) Figure 1.3. 

 Final diagnosis of metastatic bilateral adrenal sarcomatoid carcinoma was made in view of ^18^F-FDG PET/CT scan, biopsy, and IHC report. The present case died within a few months of diagnosis due to multi organ failure.

## Discussion

 Bilateral ^18^F-FDG avid adrenal lesions mimic benign (hyperplasia, pheochromocytoma, tuberculosis, histoplasmosis, myelolipoma, nontraumatic haemorrhage, vasovagal-related stress immediately before FDG injection) and malignant lesions (lymphoma, adrenocortical carcinoma (ACC), metastases) (5-8). ^18^F-FDG avid malignant adrenal tumours involving both adrenal glands are uncommon except metastatic lesions from an extra-adrenal organ, however, IHC confirmation to exclude another primary malignancy lesion is required (3). The primary malignant lesions originate from lung, gastrointestinal tract, mammary gland, kidney, and liver frequently metastasized to the adrenals (9). Adrenal biopsy is not recommended in the patient with an adrenal mass which is more likely ACC because of the high possibility of tumour spread and accurate diagnosis could be made after surgical exploration (10). In our case, final histopathological diagnosis from adrenal-ectomy specimen was not possible due to metastatic disease.

 Sarcomatoid carcinomas are tumours which contain both carcinomatous and sarcomatous differentiation. The first case of sarcomatoid cancer of adrenal origin was reported by Collina et al (11). They have been identified in a variety of organ and tissue sites, including kidney, bladder, lung, breast and oesophagus (12). 

 Middle aged patients with equal ratio in both sexes are mainly affected by primary adrenal sarcomatoid carcinoma (12). Probably due to the advanced disease at the time of diagnosis; patients diagnosed having adrenal sarcomatoid tumour have a very poor prognosis. 

 In nonmetastatic disease, adjuvant chemo-therapy is suggested after the removal of the tumour (12). Large number of patients succumbed to disease within a short interval of time after diagnosis, due to recurrence at the primary site or metastatic disease. It is essential to diagnose these tumours earliest to treat with effective treatment modalities (13). The present case died within a few months of diagnosis due to multi organ failure. The greatest chance of long term survival from adrenal sarcomatoid carcinoma to be early detection prior to metastatic spread, radical complete excision and adjuvant chemotherapy and radiotherapy in an attempt to prevent local disease recurrence (14). Always keep in mind ACC as a differential diagnosis in case of bilateral adrenal masses, as in 10% cases ACC can affect both adrenal glands (4). 
